# The effect of titanium surface treatment by application of constant potential or current on the viability of pre-osteoblast cells: an *in-vitro* study

**DOI:** 10.3389/fbioe.2024.1425450

**Published:** 2024-10-18

**Authors:** Wenji Cai, Min Wang, Amir Ei Hadad, Yuli Zhang, Simon D. Tran, Samar Shurbaji, Gheyath K. Nasrallah, Mariano Sanz, Sasha Omanovic, Faleh Tamimi

**Affiliations:** ^1^ Faculty of Dental Medicine and Oral Health Sciences, McGill University, Montreal, QC, Canada; ^2^ State Key Laboratory of Oral and Maxillofacial Reconstruction and Regeneration, Key Laboratory of Oral Biomedicine Ministry of Education, Hubei Key Laboratory of Stomatology, Wuhan, China; ^3^ Department of Oral Implantology, School and Hospital of Stomatology, Wuhan University, Wuhan, China; ^4^ College of Dental Medicine, QU Health, Qatar University, Doha, Qatar; ^5^ Department of Biomedical Science, College of Health Sciences, QU Health, Qatar University, Doha, Qatar; ^6^ ETEP Research Group, Faculty of Odontology, University Complutense of Madrid, Madrid, Spain; ^7^ Chemical Engineering department, McGill University, Montreal, QC, Canada

**Keywords:** pre-osteoblast cells, electrochemical treatment, titanium, implants, voltage, current, osteointegration

## Abstract

**Objectives:**

The aim of this study was to investigate the impact of electrochemical treatment of a titanium surface employing constant current and potential on the viability of the tissue cells attached to the surface and determining the safety limits for this type of treatment.

**Methods:**

Pre-osteoblast cells (pOB) were cultured and seeded onto titanium discs. The cell-seeded discs were then exposed to a range of contant direct electrical potentials (-6V–6V) or contant direct electrical currents (−12.5 mA, −25 mA, or −50 mA) using a three-electrode system connected to a potentiostat. Cell viability was assessed using live/dead assay and fluorescence microscopy.

**Results:**

Exposure of cells to high negative potentials caused cell detachment, while exposure to positive ones led to cell death on the cpTi surfaces. However, cellular viability was preserved when the electrical potentials were kept between −3 and +3 V. Cells retained 80% viability when subjected to −12.5 mA currents with an initial pOB cell count of 5 × 10^4^. However, when the initial cell count was elevated to 1 × 10^5^, the cells demonstrated the ability to withstand an even greater current (−25 mA) while preserving their vitality at the same level.

**Conclusion:**

Treatment of a titanium dental implant surface employing constant potential or current can harm cells surrounding dental implants. However, this damage can be minimized by keeping the potential within a safety limit.

## 1 Introduction

Peri-implantitis is an inflammatory condition affecting the tissues surrounding osseointegrated oral implants, which leads to subsequent loss of the bone providing support (Schwarz et al., 2018). This destructive inflammatory response is largely associated with bacterial biofilms that colonize the surfaces of dental implants (Mombelli and Lang, 2000). The treatment of peri-implantitis focuses on the removal of surface biofilms and re-establishing conditions conducive to osseointegration on the dental implant surfaces (Baron et al., 2000). Various strategies have been attempted for the treatment of peri-implantitis. These include the use of lasers, mechanical debridement, and chemical detoxification (Sinjab et al., 2018). This method either directly exterminate bacteria or enhance the penetration of antibiotics into the infected region (Sinjab et al., 2018). Nonetheless, some of these established techniques may inadvertently distort the implant’s surface topography or modify its properties, potentially fostering bacterial recolonization or hindering re-osseointegration (Alagl et al., 2019; Parlar et al., 2009; Larsen et al., 2017). To date, there is not a single approach that stands as a universally accepted gold standard for treatment for peri-implantitis (Renvert et al., 2008).

The bactericidal effects of electric fields and currents have been subject of research for a while, given their considerable impact on the growth or elimination of both prokaryotic and eukaryotic cells (Del Pozo et al., 2008). Electrochemical treatments have been proposed as a plausible minimally invasive method to decrease the count of viable microorganisms on dental implants (Drees et al., 2003). Mohn et al first reported the successful use of electrochemical disinfection for titanium implants contaminated with *Escherichia coli* C43 biofilm, where a mere direct current of 7.5 mA resulted in the total eradication of the bacteria (Mohn et al., 2011). The bactericidal effect of direct current (DC) seems to stem from the generation of reactive oxygen species (ROS) (Brinkman et al., 2016), and also enhance the effectiveness of antimicrobials (Costerton et al., 1994).

In a recent study published by our group we demonstrated that electrochemical treatments are effective in eliminating pathological oral biofilms within a particular range of potentials (Virto et al., 2023). We demonstrated that electrochemical treatments using potentials of +3V and -3V achieved a significant reduction in bacterial counts on dental implant surfaces, and these treatments were most effective against periodontal pathogens such as *Fusobacterium nuleatum* (Virto et al., 2023).

However, despite their benefits against bacteria, electrochemical reactions might also harm healthy tissues, and potentially cause inflammation or damage to the soft and hard tissues of the mouth (Gittens et al., 2011). Yet, the impact of electrical bactericidal treatments on the peri implant cells and tissues has not been thoroughly investigated. Indeed, in order to develop effective electrochemical treatments for peri-implantitis it is critical to identify the current and/or potential ranges that are effective in eliminating the bacteria and yet safe for the surrounding peri-implant tissues.

The aim in this study was to determine the safety limits for these electrochemical treatments to protect tissue cells adjacent to pure titanium substrates. To achieve this, we carried out this *in vitro* study to evaluate the effect of potential and current on the viability of pre-osteoblasts cells (pOB) cells growing on titanium surfaces.

## 2 Material and methods

### 2.1 Titanium sample preparation

Titanium rods (Ultra-Corrosion-Resistant Titanium, grade 2, McMaster-Carr, Cleveland, OH) were obtained and cut into smaller discs (diameter 1.6 cm and thickness 0.4 cm with surface area around 6 cm^2^) using an abrasive cutter (Delta AbrasiMet, Buchler, ON). The discs were then polished manually using silicon carbide paper (Buehler, Lake Bluff, IL, US) successively by 320, 600 and finally 1200 grit to obtain standardized surfaces. All samples were cleaned in an ultrasonic bath (FS20D Ultrasonic, Fisher Scientific, Montreal, Canada) with 100% acetone and 100% ethanol, and distilled water for 10 min each. Then, they were sterilized by autoclave (Yamato Scientific, Japan) and dried overnight in a vacuum oven.

### 2.2 Tissue cell culture

Tissue cells used in this study comprised pre-osteoblasts cells (pOB) which were obtained from a murine pre-osteoblastic cell line (MC3T3-E1 Subclone 4, ATCC^®^, CRL-2593).

Briefly, pOB cells were cultured in the Alpha Modification of Eagle’s Medium (Gibco™ MEM α, nucleosides, no ascorbic acid, Fisher Scientific, Montreal, Canada) supplemented with 10% (v/v) fetal bovine serum (Gibco™ FBS, Thermo Fisher Scientific, US) and 1% (v/v) 100X Antibiotic-Antimycotic solution (Thermo Fisher Scientific, US). After the aspiration of the culture medium, the culture dishes were rinsed with phosphate buffered saline (PBS Tablets, Gibco™) to remove serum traces from the old medium. Next, the attached cells were enzymatically freed from the dish by 2 mL 0.25% trypsin-EDTA (Gibco™). The trypsinization process was then inhibited by introducing complete culture medium to the dish. Following this, the cell solution was centrifuged, the supernatant aspirated, and the tissue cell pellet resuspended in fresh medium. Cells were enumerated using a hemocytometer in conjunction with a 0.4% trypan blue solution (Gibco™). This was performed to establish the suitable cell concentration in the cell suspension to proceed the subsequent experiments.

### 2.3 Titanium disc cell seeding

Pre-osteoblastic cells (pOB) cells were cultured onto commercially cpTi substrate before placing them into a electrochemical cell. To do that cell suspensions were prepared and 50 μL was applied onto each cpTi disc in a 12-well cell culture plate with overall cell count of either 1 × 10^5^ or 5 × 10^4^ (Sarstedt, US) and left undisturbed for 4 h to allow for cell attachment. Subsequently, 950 μL of culture medium was gently introduced to fully submerge the cpTi samples before incubation under precise environmental conditions (humidified incubator, 37°C, 5% CO_2_) for 24 h.

### 2.4 Electrochemical setup

**FIGURE 1:**
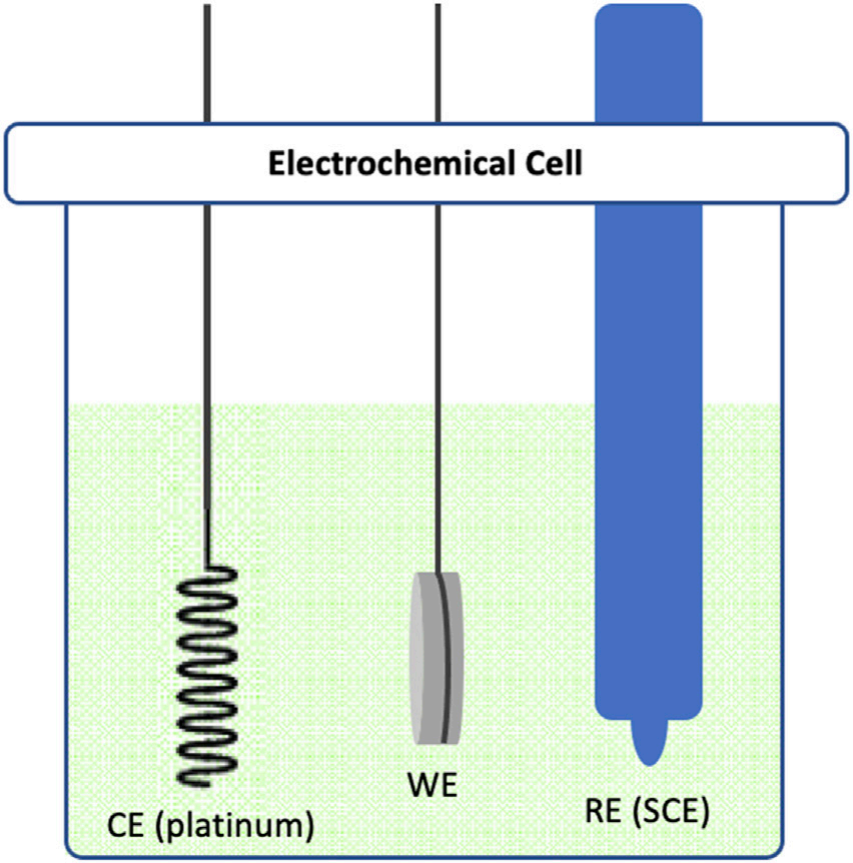
Schematic diagram illustrating the three-electrode electrochemical cell used in this study. The cell consists of a Platinum counter electrode (CE), a cpTi working electrode (WE) and a saturated calomel reference electrode (SCE).

In our experimental design, we employed a three-electrode system. Commercially pure titanium (cpTi) samples were secured by a stainless rod in a glass electrochemical cell chamber (Figure 1) interfaced with a potentiostat (VersaSTAT 4, AMETEK Scientific Instruments, US). These cpTi samples functioned as the working electrode (WE), with platinum serving as the counter electrode (CE) and a saturated calomel electrode (SCE) acting as the reference electrode (RE). The electrolyte used in the experiment was phosphate buffer (PB) without saline, maintaining a pH of 7.2. The rationale for omitting NaCl in the electrolyte was to avoid localized corrosion of cpTi surface when polarized at higher positive potentials or currents, as a consequence of chloride ions attack. A direct current (DC) was applied to the cell chamber, executing polarization either at a constant potential (−1.5V, -3V, -6V, +1.5V, +3V, or +6V) or a constant current (−12.5 mA, −25 mA or −50 mA) for 15 min. Each experiment by putting the cpTi at open circuit potential (OCP) at room temperature for 60 s, and then applying the current or potential to a specific desired value. For our control group, we utilized a similar potentiostatic chamber construct with cpTi samples in the electrolyte (PB solution), but without the application of any electrochemical treatment.

### 2.5 Cell viability

The viability and morphological assessment of cells post-electrochemical treatment were carried out using the live/dead staining procedure following the ISO 10993-5 protocol (Neuss et al.). Staining solutions were prepared by mixing 2 μL fluorescein diacetate (FDA, λmax/λem = 490/514 nm, Invitrogen, Fisher Scientific) (5mg/1m acetone), 2 μL of propidium iodide (2 mg/1 mL PBS.) (PI, λmax/λem = 490/620 nm, Invitrogen) and 1 mL of culture medium. After undergoing the electrochemical treatments, samples were washed with PBS for removal of the culture media, and subsequently incubated in the prepared staining solutions for 5 min. Then, samples were subjected to another round of PBS wash before proceeding with image acquisition.

Cells were analyzed with the Zeiss AX 10 fluorescence microscope (Carl Zeiss, Göttingen, Germany) and three random images were obtained from each sample using the ×10 objective lens. The complete view of the cpTi surface was also obtained using the tiles tool. Green fluorescence indicates viable cells while red indicates dead cells. These images were segmented and the surface-adherent tissue cells were counted automatically using the Fiji ImageJ2 package (Version 1.53f) (Schindelin et al., 2012).

### 2.6 Statistical analysis

All experiments were performed in triplicates (n = 3). One-sample Kolmogorov-Smirnov and Shapiro-Wilk tests were conducted to ascertain the normal distribution of the data. For normally distributed data significant differences across groups were assessed using one-way ANOVA with Tukey’s *post hoc* analysis was used, with a significance level set at *p* < 0.05. Unpaired two samples *t*-test were also used for the comparison between two groups. IBM SPSS Statistics 29.0 (IBM Corporation, Armonk, NY, USA) was employed for all statistical evaluations.

## 3 Results

### 3.1 Treatment of pOB-covered cpTi at constant current

**FIGURE 2:**
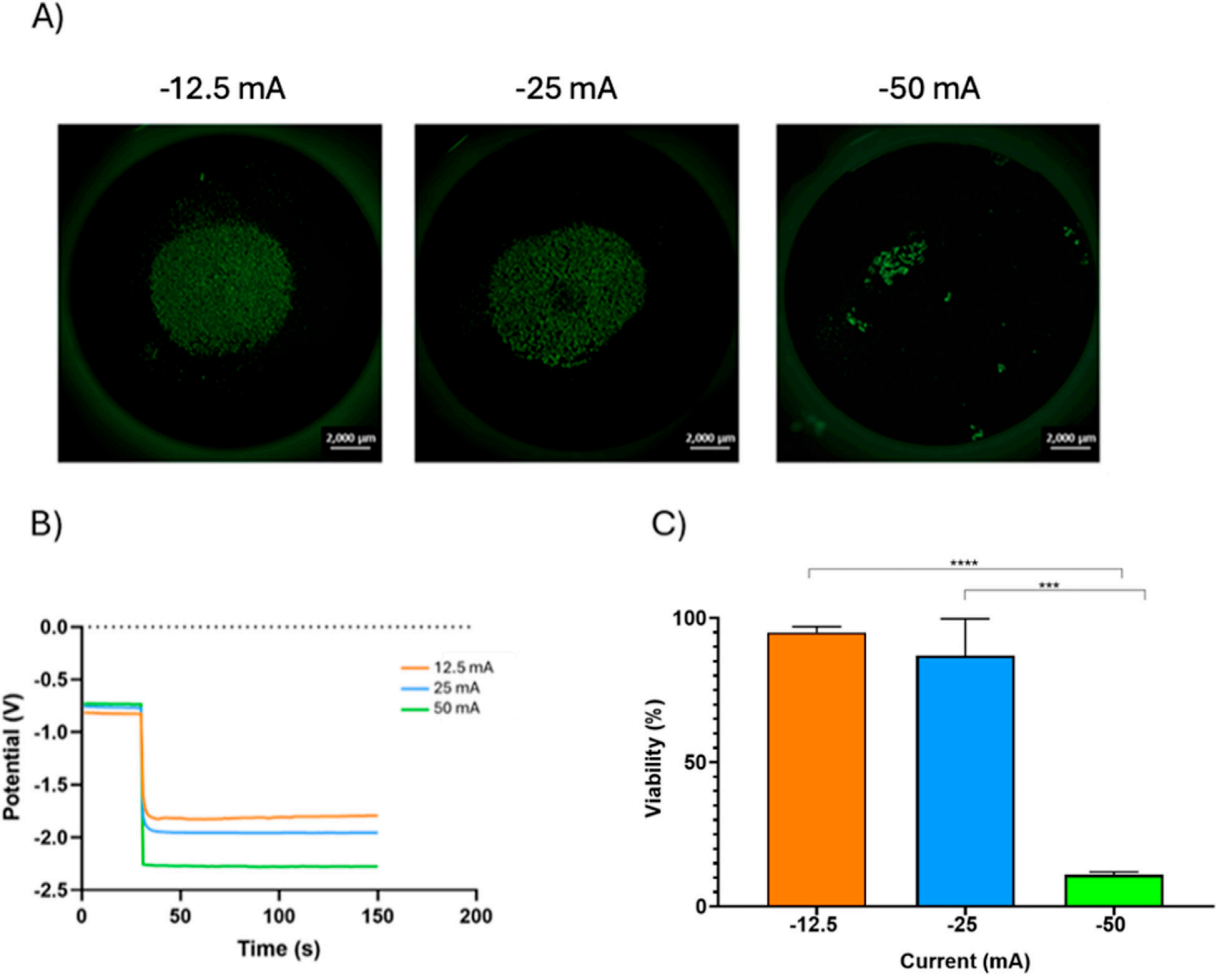
The effect of different currents on the viability of POB cells with 1 × 10^5^ concentration. **(A)** Fluorescence microscopy images of the live/dead POB cells on the cpTi disc recorded after the electrochemical galvanostatic treatment (scale bar = 2000 µm). **(B)**Potential-time curves portray the effects when varying direct currents were imposed on cpTi coated with 1 × 10^5^ POB cells. **(C)** relationship between current applied and cell viability.

Due to the well documented ability of negative electrical currents to remove contaminants from Ti surfaces we investigated the effect of fixed negative currents on cpTi seeded with tissue cells. Higher currents (−50 mA) generated a more negative potential (−2.25V) on pOB seeded cpTi, while lower currents (-12.5 mA) yielded less negative potentials (−1.75V) (Figures 2A, B).

Initial cell concentration had little effect on the potentials generated by the different currents. There was a direct relation between post treatment cell count and the electrical currents; higher currents (−50 mA) were more effective in reducing cell counts than lower currents (−12.5 mA). Interestingly, higher initial cell density seemed to have a protective effect against the currents; samples with higher initial cell counts presented a higher percentage of cell vitality after exposure to the electrical currents than those with lower initial cell counts. An 80% cell vitality was maintained for cells exposed to currents of −12.5 mA when the initial pOB cell count was 5 × 10^4^, but cells were able to tolerate an even higher current (−25 mA) while maintain the same vitality when the initial cell count was higher (1 × 10^5^) (Supplementary Figure S1). In general, as the consequence of the electrochemical treatment at constant current, the viability of the pOB cells on the cpTi surface decreased with an increase in negative current, as seen in Figure 2C. At −12.5 mA the viability of the pOB cells after the treatment was 95 %, and it decreased slightly to 87% at −25 mA. However, when the treatment was done at −50 mA, the cell viability dropped significantly, to only 11%.

### 3.2 Treatment of pOB-covered cpTi at constant potential

**FIGURE 3:**
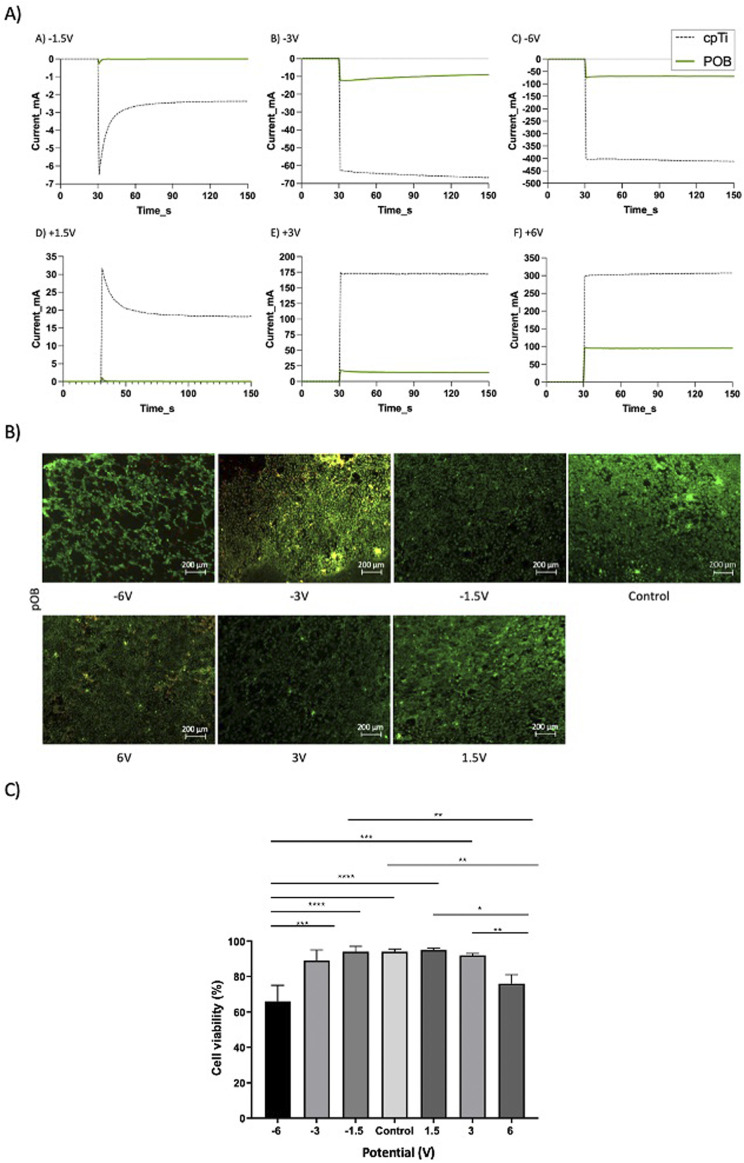
**(A)** Current-time curves recorded at different electrode potentials on the naked cpTi surface (gray dotted line) and the cpTi surface coated with 1x105 pOB (green line), repeated three times. The samples were kept at an open circuit potential (OCP) for the first 30 seconds, upon which a constant potential was applied; **(B)** Fluorescence microscopy images of the live/dead pOB on a cpTi disc recorded after the potentiostatic electrochemical treatment at various potentials (scale bar = 200μm); **(C)** Bars plots showing cell viability for the pOB remained on the cpTi surface at post-treatment. All the samples, except for control group, were kept at an OCP for the first 30 seconds, upon which a constant potential was applied for 2 min. * in the bar charts indicates significant differences.

The results in Figure 2 showed that by treating a cpTi surface covered by pOB cells at a constant current can result in retention of cell viability at low currents, but also in a significant decrease in cell viability at high negative currents (-50 mA). At this latter current the resulting cpTi surface potential was -2.25 V (vs. SCE). However, it was also of interest to investigate the influence of electrochemical treatment of the pOB-covered cpTi surface at a constant potential. With this in aim, the surface-treatment experiments were performed from -6 V to 6 V, and the control experiment was performed at open circuit. The resulting current-time curves are presented in Figure 3A as green solid lines. As control, the current-time curves were also recorded in the absence of cells on the surface (dashed black curves). As expected, with an increase in treatment potential in both directions with the naked surface, the resulting current increases due to the occurrence of the hydrogen (negative potentials) and oxygen (positive potentials) evolution reactions (dashed curves). When the cells are present on the surface, the resulting current is significantly lower at all potentials (green curves), although it also increases when the surface is polarized in both directions. The lower current recorded is due to the blockage of the metal substrate surface with the cells, minimizing exposure of the surface to the electrolyte and thus, consequentially, minimizing the rate of hydrogen and oxygen evolution reactions.

Figure 3B indicates that the cell surface density is not influenced much when the cpTI/pOB surface was treated between -3V and 6V. However, at -6V, a noticeable decrease in cell surface count was noted.

**FIGURE 4:**
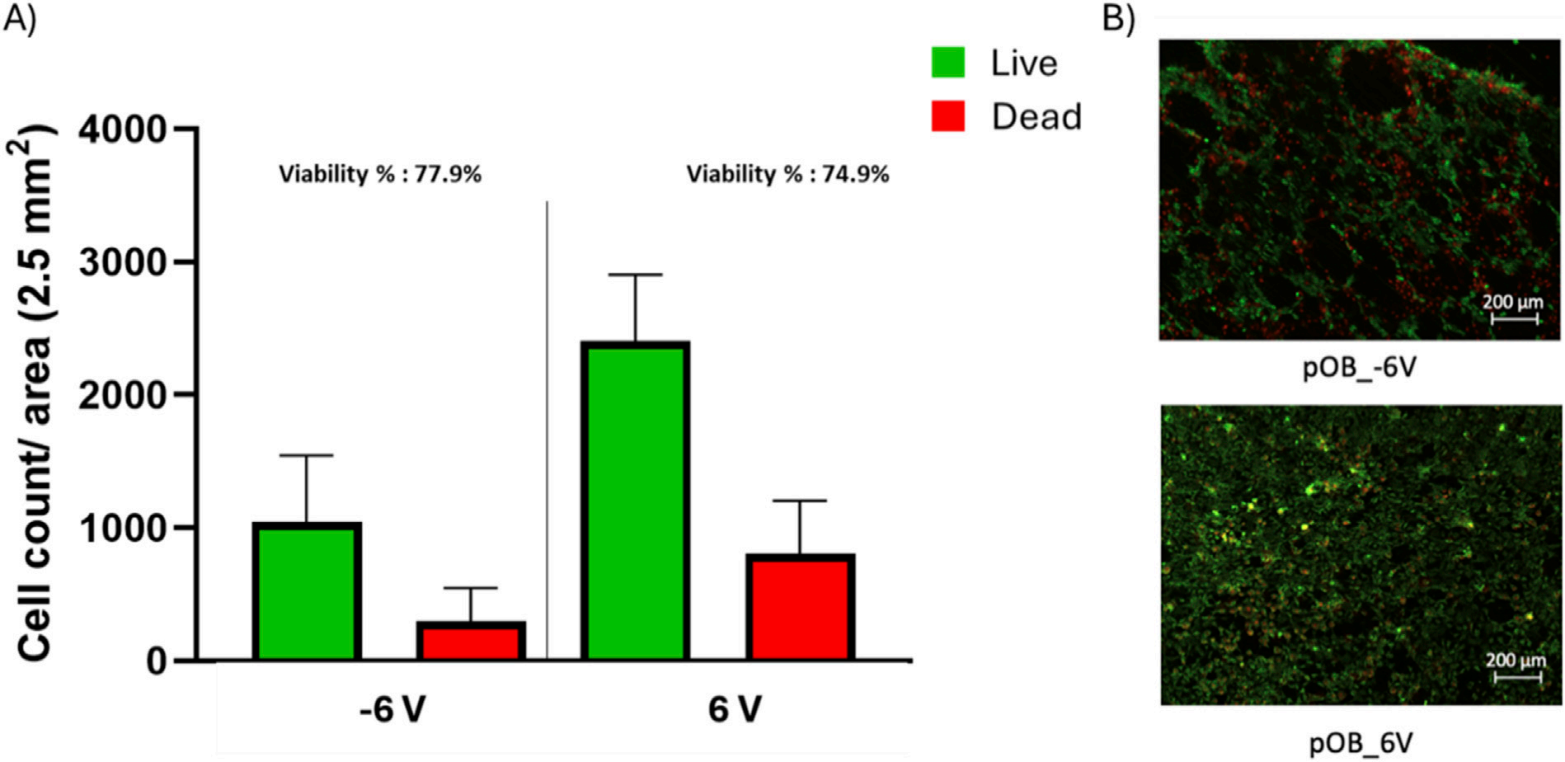
Post-treatment live/dead assay of pOB on a cpTi surface. **(A)** bar chart representing live and dead cell count for both −6 and 6 V. **(B)** live/dead staining for poB cells exposed to −6 and 6 V, green represents the live cells while read represents the dead cells. Scale bar = 200 μm.

The live/dead assay revealed that the highest levels of cell viability were obtained between -1.5V and +1.5V, with only a slight drop in cell viability at -3V and +3V (Figures 3B, C). However, the treatment at high negative potentials (-6V) resulted in significant decrease in cell viability (Figure 3C) and cell detachment (Figure 3B and Figure 4). Although there is no significant difference between the viability percentage in the -6 V and 6 V groups (77.9% and 74.9 % respectively, Figure 3C), the cell count (Figure 4) was found to be double when the surface was treated at the positive potential. The results indicate cell's detachment in the negative potential group rather than cell death.

In summary, applying different potentials to pOB-seeded cpTi substrates for 2 minutes revealed a safe range of electrochemical surface treatment between -3V to 3V, resulting in a higher cell viability.

## 4 Discussion

In this study, we showed how electrical currents and potentials on titanium surfaces interact with pre-osteoblasts cells on the metal surface. MC3T3-E1 Subclone 4 preosteoblasts were isolated from the cloned but phenotypically heterogeneous MC3T3-E1 cell line. This subclone was selected for high osteoblast differentiation and mineralization after growth in medium containing ascorbic acid. This cell line is a good model for studying *in vitro* osteoblast differentiation, particularly ECM signaling. It's behavior is similar to primary calvarial osteoblasts. Our findings showed that while high-intensity electrical currents such as −50 mA and potentials could cause cell detachment and death, there is a safe range of currents and potentials within which the cells remain viable on the surface.

Both negative and positive potentials caused cell detachment. This could be due to the production of hydrogen and oxygen bubbles on the surface that occurred with both positive and negative potentials.

Negative potentials result in a reduction process that produces hydrogen gas (H_2_) bubbles when the titanium surface acts as a working cathode:

$${2H}_{2}O+{2e}^{-}\to H_{2}+2OH^{-}$$

Conversely, when a positive current is applied to the cpTi, it becomes an anode in the electrochemical cell and undergoes oxidation resulting in the production of oxygen (O_2_) bubbles:

$$2H_{2}O\to O_{2}+4H^{+}+4e^{-}$$

Both negative and positive potentials formed bubbles and caused cell detachment, however this was more apparent with negative potentials. This phenomenon can be explained by the presence of titanium dioxide (TiO_2_) on the cpTi surface. Titanium dioxide, is an n-type semiconductor with unique electrochemical characteristics that influence the gas evolution behavior under different potentials (Nowotny et al., 2007). For example, the TiO_2_ film does not pose significant electrical resistance to negative potentials, facilitating H_2_ evolution. However, with positive potentials, the n-type semiconductor characteristics of the TiO_2_ film plays a different role. Namely, when the cpTi surface is positively polarized a large portion of the potential drop is within the (resistive) oxide film, while the potential drop on the negatively-polarized cpTi surface is within the electrochemical double layer; consequently a significantly larger portion of the driving force (electrode potential) applied is used to evolve hydrogen than that to evolve oxygen. This is also affirmed by the lower absolute value of current observed when bare cpTi is under positive polarization, compared to when it's under negative potential. The diminished current in turn yields a smaller rate of cell removal from the surface.

Regarding cell vitality, positive potentials were more likely to cause cell death, although high voltage negative potential also resulted in low vitality. This seems to indicate that reductive environment caused by negative potentials seem to be less harmful to cell vitality than the oxidizing conditions generated at the anode (Pizzino et al., 1942-0994). In fact, in some circumstances, small amounts of hydrogen gas produced by negative potentials can even have cytoprotective effects (Ge et al.). In this sense exposure to a potential between −1.5V and +1.5V seems to be the safest for pOB cells since the highest levels of cell vitality were maintained between these levels.

Cell vitality depended on cell surface density and the electrical currents. Higher cell dentisty seem to protect pOB cells from death by electrical currents. The current should not exceed approximately −12.5 mA for a pOB cell density of 5 × 10^4^ and around −25 mA for the density of 1 × 10^5^. External electric field has been demonstrated to modulate cellular biomechanical properties such as microfilament reorganization (Li and Kolega)or alterations in transmembrane calcium dynamics (Cho et al.), thereby providing potential avenues for controlling cell metabolism.

Our investigations suggest an absolute safety range of -3V for pOB cells for 2min DC stimulation, fortuitously aligning with electrochemical parameters for biofilm removal reported by other researchers. In particular, electrochemical treatments at both 3V and -3V were observed to significantly diminish total bacterial counts (Virto et al., 2023). Notably, the -3V treatment yielded a stronger antibacterial effect, especially against the bacterium *Fusobacterium nucleatum*. In contrast, the biofilm remained largely unaffected by the treatments at 0.75V and −0.75V (Virto et al., 2023). Additionally, the application of cathodic voltage stimulations at −1.8V on titanium surfaces has been reported to effectively inhibit the colonization by Gram-positive methicillin-resistant *Staphylococcus aureus* and Gram-negative *Acinetobacter baumannii* (Canty et al., 2017). This collection of findings provides compelling insights into potential voltage parameters for both safe cellular function and effective antibacterial action.

In addition, the chronopotentiometry experiment showed that a greater initial cell density led to two main outcomes. First, for a given electrical current, these denser populations exhibited lower voltages, suggesting potential resistance to voltage fluctuations. Second, they experienced less cell loss after the electrochemical procedure, indicating higher cell viability. Thus, the starting cell density significantly influences polarization during the constant-current treatment chronopotentiometry, affecting not just voltage response and cell survival, but also determining the safe limit for applied currents. The existing literature concerning cells cultured directly on titanium has largely overlooked the influence of initial cell density on electrical resistance and cellular behavior (Ehrensberger et al., 2010; Haeri et al., 2013; Bishal et al., 2017). According to the results of our current investigation, these electrochemical variables could considerably impact cell viability. Hence, these findings underscore the necessity to incorporate these factors into subsequent experimental designs.

This study presents certain limitations, one of which is the established timeframe for polarization. The efficacy of the electrochemical treatment in diminishing bacterial colonization has demonstrated a dependency on not only the magnitude but also the duration of the polarization Mohn et al., 2011. The selected polarization timeframe in this study was benchmarked against previous literature focused on biofilm removal (Sahrmann et al., 2014; Schneider et al., 2018; Ratka et al., 2019). Secondly, the *in vitro* model used might not accurately represent the oral cavity’s complex environment due to potential discrepancies in essential nutrients, physicochemical conditions such as pH and flow conditions. These factors need to be taken into consideration for future research to ensure a more comprehensive understanding of the phenomena. Additionally, various advanced surface modification techniques have been employed to improve the osseointegration of dental implants, such as additive surface coatings with active ions and subtractive surface anodic oxidation (Meng et al., 2022). These different methods might require tailored electrochemical settings for effective surface decontamination. Further, the research to date has primarily focused on the bactericidal effects of direct current (DC), leaving the potential impact of alternating current (AC) relatively unexplored (Virto et al., 2023). Numerous studies have hinted at the superior efficacy of alternating electric fields for biofilm removal while safeguarding tissue cells. Several reports have suggested the better application of alternative electric fields in the biofilm removal while preserving tissue cells. Specifically, an alternating current within 1.5V appears to positively stimulate osteogenic gene expression and alkaline phosphatase activity, whereas levels over 2.8V may produce detrimental effects (Sahm et al., 2020). An alternating current (−2.3 mA, + 22.5 μA) and a voltage of 1.8 V have demonstrated bactericidal properties, effectively decontaminating saliva-contaminated titanium surfaces in 5 min while maintaining surface integrity and histological quality of mammalian tissues (Al-Hashedi et al., 2016).

In conclusion, this present investigation provides valuable insights into the effects of potentials and currents on pOB cells attached on a cpTi surface. The treatment of the surface at a constant high negative potential result in cell detachment, while the treatment at a high positive potential leads to cell death on cpTi surfaces. The presence of a TiO_2_ film on the cpTi surface influences gas evolution under different potentials. Future studies should incorporate such as X-ray Photoelectron Spectroscopy (XPS) analyses to precisely determine the surface composition of titanium samples subjected to electrochemical treatments. This will provide a more robust basis for understanding the interactions between the electrochemical environment and cell viability. The initial cell count plays a significant role in polarization, voltage response, cell survival, and the safe limit for applied currents. These findings emphasize the importance of considering initial cell count and electrochemical variables when studying cell-substrate interactions and electrochemical dynamics.

## Data Availability

The original contributions presented in the study are included in the article/Supplementary Material, further inquiries can be directed to the corresponding author.
