# Supplementary material for: The effect of titanium surface treatment by application of constant potential or current on the viability of pre-osteoblast cells: an in-vitro study
Source: Front Bioeng Biotechnol. 2024 Oct 18;12:1425450. doi: 10.3389/fbioe.2024.1425450 (PMC11527693; doi:10.3389/fbioe.2024.1425450)
Supplement: Supplementary file 1 [file DataSheet1.docx]

**Supplementary material**

**Safety of Electrochemical decontamination of titanium on Preosteoblasts: an in vitro Study**

Wenji Cai^1^, Min Wang^2^, Amir EI Hadad^1^, Yuli Zhang^1^, Simon Tran^1^, Samar Shurbaji^5^, Gheyath Nasrallah^6^, Mariano Sanz^3^, Sasha Omanovic^4^, Faleh Tamimi^5^

^1^Faculty of Dental Medicine and Oral Health Sciences, McGill University, Montreal, Canada

^2.^ State Key Laboratory of Oral & Maxillofacial Reconstruction and Regeneration, Key Laboratory of Oral Biomedicine Ministry of Education, Hubei Key Laboratory of Stomatology; Department of Oral Implantology, School & Hospital of Stomatology, Wuhan University.

^3^ETEP Research Group, Faculty of Odontology, University Complutense of Madrid, Madrid, Spain

^4^Chemical Engineering department, McGill University, Montreal, Canada

^5^College of Dental Medicine, QU Health, Qatar University, Doha, Qatar

^6^Department of Biomedical Science, College of Health Sciences, Member of QU Health, Qatar University, 2713, Doha, Qatar.

^*^Authors to whom correspondence should be addressed.

^*^Corresponding author:fmarino@qu.edu.qa, +974 4403 7307


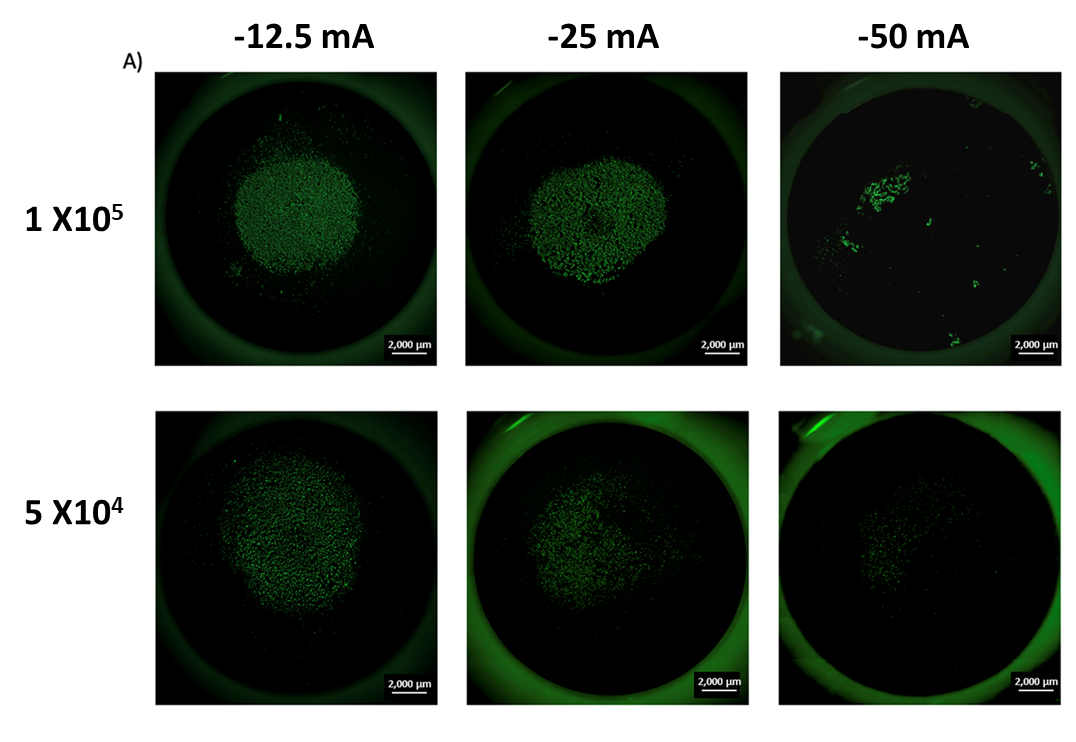


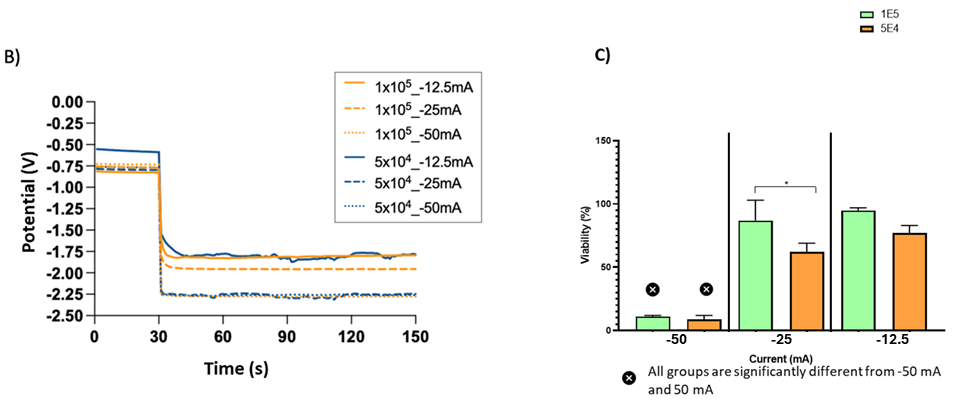


**Fig 1.** A) Fluorescence microscopy images of the live/dead POB cells on the cpTi disc recorded after the electrochemical galvanostatic treatment (scale bar = 2000µm). The POB cell count seeded onto the titanium was either 1x10^5^ or 5x10^4^ per cpTi, and the applied constant direct current was -12.5mA, -25mA, or -50mA; B) Potential-time curves portray the effects when varying direct currents were imposed on cpTi coated with 1x10^5^ POB cell counts (orange line) or cpTi coated with 5x10^4^ POB cell counts (blue line). Each galvanostatic pulse commenced after keeping the cpTi sample at OCP for 30s. C) Relationship between the cell counts of surviving pOB on the cpTi and the corresponding current applied during the electrochemical surface treatment The number of alive cells that remained on the cpTi surfaces was significantly higher in the 1x10^5^ group.
